# Maternal and Neonatal Outcomes in Gestational Hypertension for Delivery at 37 versus 38 to 40 Weeks

**DOI:** 10.1055/a-2568-9104

**Published:** 2025-04-17

**Authors:** Lauren Thompson, Joseph Werthammer, Grace Montgomery, Matthew Nudelman, Jesse Cottrell, David Gozal, Rebekah Fabela, Kennedy Snavely

**Affiliations:** 1 Division of Neonatal-Perinatal Medicine, Department of Pediatrics, Marshall University Joan C. Edwards School of Medicine, Huntington, West Virginia; 2 Department of Obstetrics and Gynecology, Marshall University Joan C. Edwards School of Medicine, Huntington, West Virginia; 3 Section of Applied Clinical Informatics, Department of Medicine, University of Wisconsin School of Medicine and Public Health, Madison, Wisconsin; 4 Division of Maternal-Fetal Medicine, Department of Obstetrics and Gynecology, Marshall University Joan C. Edwards School of Medicine, Huntington, West Virginia; 5 Department of Pediatrics, Marshall University Joan C. Edwards School of Medicine, Huntington, West Virginia; 6 Marshall University Joan C. Edwards School of Medicine, Huntington, West Virginia

**Keywords:** gestational hypertension, timing of delivery, preeclampsia, maternal hypertensive disorders, adverse perinatal outcomes

## Abstract

**Objective:**

This study aimed to compare neonatal and maternal outcomes for mothers with gestational hypertension delivered at 37 weeks' gestation compared with 38 to 40 weeks.

**Study Design:**

Single-center, retrospective chart review of women with gestational hypertension delivered between 37 ^0/7^ and 40 ^6/7^ weeks' gestation over a 29-month period.

**Results:**

A total of 337 mother–infant dyads with gestational hypertension were included: 194 delivered at 37 weeks' gestation (cohort 1) and 143 delivered at 38 to 40 weeks' gestation (cohort 2). Preeclampsia developed in 12% of cohort 1 and 8% of cohort 2 ( *p* = 0.242). No significant differences in severe hypertensive-related complications were found between the cohorts. Neonatal outcomes including neonatal intensive care unit admission, respiratory support, phototherapy, and length of stay were all more frequent in cohort 1.

**Conclusion:**

For women with gestational hypertension, delivery at 38 to 40 weeks was not associated with increased maternal complications but was associated with fewer neonatal complications when compared with delivery at 37 weeks.

**Key Points:**

- Gestational hypertension is a common complication of pregnancy.
- Mothers with gestational hypertension are often induced early.
- Neonatal adverse outcomes increase with early delivery.
- Optimal timing of delivery for pregnancies complicated by gestational hypertension is unknown.

Hypertensive disorders during pregnancy are characterized by elevated blood pressure (BP) during the antenatal period and include gestational hypertension, chronic hypertension, preeclampsia, preeclampsia superimposed on chronic hypertension, and eclampsia. 1 More than 10% of pregnancies are complicated by hypertensive disorders, with increasing prevalence in the United States over recent years. 2 3 4 These disorders carry risks for both the mother and baby, and the only effective treatment is delivery. 5 These disorders are accompanied by substantial maternal and neonatal morbidity and mortality worldwide. 6 In high-income countries, 10 to 16% of maternal deaths during pregnancy can be attributed to hypertensive disorders. 5 6 7 8 9

Gestational hypertension is defined as a systolic BP ≥140 and <160 mm Hg and/or a diastolic BP of ≥90 and <110 mm Hg on two occasions at least 4-hour apart after 20 weeks' gestation, in a woman who was previously normotensive. 10 Women with gestational hypertension progress to mild preeclampsia at a rate of up to 46% and to severe preeclampsia at 9.6%. 11 Controversy exists as to the optimal timing for the delivery of mothers with gestational hypertension when aiming to balance the fetal benefits of expectant management with the maternal and fetal risks associated with early delivery. For instance, the American College of Obstetrics and Gynecology recommends delivery of pregnant women with uncomplicated gestational hypertension at 37 ^0/7^ weeks' gestation, 12 whereas the International Society for the Study of Hypertension in Pregnancy suggests expectant management up to 39 ^6/7^ weeks' gestation for pregnancies with well-controlled BP, if there is reassuring evidence regarding fetal status and no signs of preeclampsia. 13 The objective of this study was to compare maternal and fetal outcomes for mothers who delivered at 37 weeks versus those who delivered from 38 to 40 completed pregnancy weeks.

## Materials and Methods

This was a retrospective cohort study from a single tertiary care perinatal center of women giving birth from October 1, 2020, to February 28, 2023. The study was based at Cabell Huntington Hospital in Huntington, West Virginia, the perinatal teaching hospital for the Marshall University Joan C. Edwards School of Medicine. An in-house attending obstetrician and resident team were available 24 hours per day. Antepartum, intrapartum, and postpartum patient information were collected following delivery using the Cabell Huntington Hospital Clinical Data Warehouse. Of the 2,962 total deliveries, 586 were coded for the diagnosis of gestational hypertension. Obstetrical and neonatal records were manually reviewed, and data extracted and recorded in a secure REDCap database. For this study, gestational hypertension was defined as systolic BP ≥140 and <160 mm Hg and/or a diastolic BP of ≥90 and <110 mm Hg confirmed by two measurements at least 4 hours apart after 20 weeks' gestation. All obstetricians used the same definition of gestational hypertension. Only singleton pregnancies were included in this study. Mothers with previously known chronic hypertension, nonthyroid autoimmune disease, and those late to prenatal care (after 14 weeks) or with incomplete prenatal records were excluded from this study. Neonates with genetic disorders and those requiring prolonged hospital stays due to neonatal abstinence syndrome and congenital syphilis were also excluded.

Maternal outcomes included preeclampsia, the need for antihypertensive medication, hospital readmission within 14 days after discharge due to elevated BP or BP-related complications, primary cesarean section, length of hospitalization, and other severe hypertensive-related complications including placental abruption, eclampsia, severe hypertension (BP ≥ 160/≥110 mm Hg), pulmonary edema, HELLP (Hemolysis, Elevated Liver enzymes and Low Platelets) syndrome, renal insufficiency (creatinine > 1.1 mg/dL), thrombocytopenia (platelet count < 100,000/mL), liver dysfunction (liver enzymes >2× upper limits and/or severe right upper quadrant abdominal pain), visual disturbances, severe headache unresponsive to medication, stroke, myocardial infarction, and death.

Neonatal outcomes included neonatal intensive care unit (NICU) admission, hospital length of stay, respiratory support after NICU admission (high-flow nasal cannula, nasal continuous positive airway pressure, synchronized inspiratory positive airway pressure, RAM cannula, and/or mechanical ventilation), need for phototherapy, small for gestational age (birth weight < 10%), exclusive breastfeeding at discharge, and death.

The protocol for this study was approved by Marshall University's Institutional Review Board (study protocol: 1910767-7). The need for informed consent was waived due to the retrospective nature of the study.

### Statistical Analysis

Categorical variables were compared by chi-square or Fisher's exact tests. Continuous variables were analyzed using univariable linear and quantile regression. A two-tailed *p* -value of <0.05 was considered significant for all tests. Statistical analysis was performed using STATA (Statacorp. 2022 Stata Statistical Software: Release 17. College Station, TX).

## Results

**Fig. 1:**
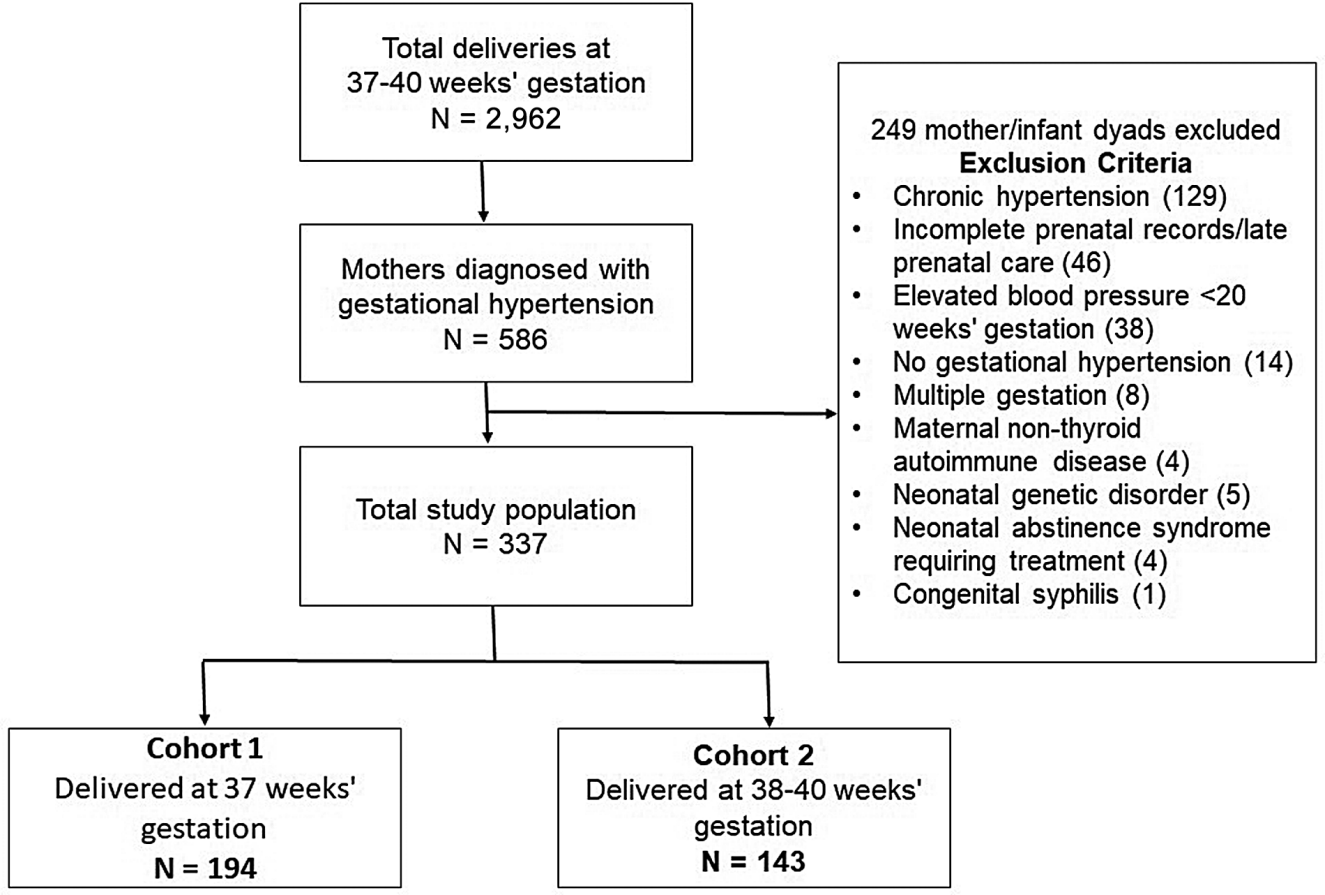
Flowchart of study participants.

A total of 2,962 pregnancies lasting between 37 ^0/7^ and 40 ^6/7^ weeks' gestation occurred during the study period. Of these, 586 were coded for the diagnosis of gestational hypertension. After exclusions, gestational hypertension was confirmed in 337 (11.4%) of these pregnancies: 194 in cohort 1 and 143 in cohort 2 ( Fig. 1 ).

### Maternal Characteristics

Maternal characteristics and demographics are shown in Table 1 . Most mothers were Caucasian and were of similar age and body mass index at the time of delivery. There were no statistical differences between the cohorts regarding rates of gestational diabetes, type II diabetes, and thyroid disease. There was no statistical difference between cohorts for the highest mean systolic BP or the highest mean diastolic BP during hospital admission for delivery.

**Table 1:** Maternal characteristics

	37 weeks' gestation *N* = 194	38–40 weeks' gestation *N* = 143	Difference (95% CI)	*p* -Value
Maternal age at delivery, mean (SD)	27 (5)	26 (5)	−1 (−2, 0)	0.151
Delivery body mass index, mean (SD)	37 (8)	36 (8)	−2 (−3, 0)	0.126
Systolic blood pressure at admission for delivery, mean (SD)	134 (12)	135 (12)	2 (−1, 4)	0.239
Diastolic blood pressures at admission for delivery, mean (SD)	82 (10)	84 (9)	2 (0, 4)	0.085
Highest systolic blood pressure during admission, mean (SD)	152 (14)	154 (16)	1 (−2, 5)	0.466
Highest diastolic blood pressure during admission, mean (SD)	90 (14)	87 (11)	−2 (−5, 1)	0.138
Public health insurance	99/194 (51%)	68/143 (48%)	−3% (−8, 2)	0.252
Race/ethnicity White alone, non-Hispanic	187/194 (96%)	134/143 (94%)	−3% (−8, 2)	0.252
Gestational diabetes	19/194 (10%)	13/143 (9%)	−1% (−7, 6)	0.828
Type II diabetes	3/194 (2%)	0/143 (0%)	−2% (−3, 0)	0.265
Thyroid disease	14/194 (7%)	7/143 (5%)	−2% (−7, 3)	0.384

Abbreviations: CI, confidence interval; SD, standard deviation.

### Maternal Outcomes

There were no maternal deaths or instances of stroke, myocardial infarction, HELLP (haemolysis, elevated liver enzymes, low platelet count) syndrome, eclampsia, or renal insufficiency in either cohort. There were no statistical differences in maternal onset of preeclampsia, need for antihypertensive medication, hospital readmission, severe hypertensive-related complications, or length of hospitalization between cohorts ( Table 2 ). The rate of cesarean section and primary cesarean section were similar between the two cohorts ( Table 3 ). However, repeat cesarean sections occurred at a significantly higher rate in cohort 1 (18 vs. 6%, *p* = 0.001).

**Table 2:** Maternal outcomes

	37 weeks' gestation *N* = 194	38–40 weeks' gestation *N* = 143	Difference (95% CI)	*p* -Value
Cesarean section	60/194 (31%)	38/194 (27%)	−4% (−14,5)	0.384
Induction due to gestational hypertension	133/194 (69%)	62/143 (43%)	−26% (−40,15)	<0.001
Preeclampsia	24/194 (12%)	12/143 (8%)	−4% (−11,3)	0.242
Uncontrolled, severe range blood pressure during delivery (>160 systolic and/or >110 diastolic)	13/194 (7%)	4/143 (3%)	−4% (−8,1)	0.133
Required blood pressure medication during or after delivery	20/194 (10%)	16/143 (11%)	1% (−6,8)	0.796
Severe hypertension-related complication a				
Placental abruption	2/194 (1%)	1/143 (1%)	0% (−2,2)	1
Pulmonary edema	1/194 (1%)	0/143 (0%)	−1% (−2,1)	1
Thrombocytopenia b	2/185 (1%)	1/141 (1%)	0% (−2,2)	0.264
Liver dysfunction c	2/177 (1%)	2/121 (2%)	1% (−2,3)	0.070
Visual disturbances	5/194 (3%)	0/143 (0%)	−3% (−5,0)	0.075
Severe headache	1/194 (1%)	2/143 (1%)	1% (−1,3)	0.577
Length of stay in days, mean (SD)	3 (1)	3 (1)	0 (0,0)	0.047
Hospital readmission within 2 wk of delivery	7/194 (4%)	3/143 (2%)	−2% (−5,2)	0.527

Abbreviations: CI, confidence interval; HELLP, Hemolysis, Elevated Liver enzymes and Low Platelets; SD, standard deviation.

a There were no instances of stroke, myocardial infarction, HELLP syndrome, eclampsia, renal insufficiency (creatinine > 1.1 mg/dL), or death in either group.

b Thrombocytopenia is defined as platelets < 100,000/mL.

c Liver dysfunction is defined as aspartate aminotransferase and/or alanine aminotransferase ≥2× the upper limit of normal or severe right upper quadrant pain.

**Table 3:** Cesarean section rates

	37 weeks' gestation *N* = 194	38–40 weeks' gestation *N* = 143	Difference (95% CI)	*p* -Value
Cesarean sections, total	60/194 (31%)	38/143 (27%)	−4% (−14, 5)	0.384
Primary cesarean section	26/194 (13%)	30/143 (21%)	8% (−1, 16)	0.065
Repeat cesarean section	34/194 (18%)	8/143 (6%)	−12% (−18, −5)	0.001
Cesarean indication				
Failed induction	28/194 (14%)	23/143 (16%)	2% (−6, 9)	0.676
CPD	0/194 (0%)	1/143 (1%)	1% (−1, 2)	0.243
Fetal distress	1/194 (1%)	1/143 (1%)	0% (−2, 2)	0.828
Elective primary	3/194 (2%)	0/143 (0%)	−2% (−3, 0)	0.135
Elective repeat	25/194 (13%)	8/143 (6%)	−7% (−13, −1)	0.026
Breech presentation	1/194 (1%)	5/143 (3%)	3% (0, 6)	0.041
Other	2/194 (1%)	0/143 (0%)	−1% (−2, 0)	0.223

Abbreviations: CI, confidence interval; CPD, cephalopelvic disproportion.

### Neonatal Characteristics and Outcomes

Neonatal characteristics and outcomes are shown in Table 4 . The median gestational age in cohort 1 was 37.0 versus 39.0 weeks' gestation in cohort 2. There was a similar percentage of male infants in each cohort. Infants in cohort 1 required admission to the NICU at significantly higher rates (14 vs. 6%, *p* = 0.025) and had a longer length of stay when admitted to the NICU (6 vs. 2 days, *p* = 0.049) compared with those in cohort 2. Infants delivered at 37 weeks' gestation also required more respiratory support and phototherapy and had lower rates of exclusive breast feeding at discharge compared with those delivered at 38 to 40 weeks' gestation. The percentage of small for gestational age infants was similar in both cohorts (4 vs. 3%, *p* -value = not significant).

**Table 4:** Neonatal outcomes

	37 weeks' gestation *N* = 194	38–40 weeks' gestation *N* = 143	Difference (95% CI)	*p* -Value
Neonatal sex, male	111/194 (57%)	76/143 (53%)	−4% (−15, 7)	0.457
Gestational age, wk, median (IQR)	37.0 (37.0,37.1)	39.0 (39.0,39.4)	1.9 (1.6, 2.0)	<0.001
NICU admission	27/194 (14%)	9/143 (6%)	−8% (−14, −1)	0.025
Respiratory support	17/194 (9%)	2/143 (2%)	−12% (−18, −7)	<0.001
Exclusive breastfeeding at discharge	122/192 (64%)	106/142 (75%)	11% (1, 21)	0.093
Birth weight, g, mean (SD)	3,174 (394)	3,442 (409)	268 (181, 355)	<0.001
Small for gestational age, birth weight < 10th percentile	7/194 (4%)	4/143 (3%)	−1% (−5, 3)	0.687
NICU length of stay, d, median (IQR)	6 (3, 10)	2 (2, 4)	−4 (−8, 0)	0.049

Abbreviations: CI, confidence interval; IQR, interquartile range; NICU, neonatal intensive care unit; SD, standard deviation.

## Discussion

In the context of our routine clinical practice, we have been steadily concerned by the rising frequency of admissions to the NICU of infants born at 37 weeks' gestational age manifesting complications of late prematurity. Many of these infants are delivered by induction of labor or repeat cesarean section solely for maternal gestational hypertension. In parallel, we are also keenly aware of the sizable proportion of mothers with gestational hypertension who delivered at 38 to 40 weeks and were discharged home with their babies without any significant complications. The dichotomous impressions generated by these trends were the impetus for our study.

In the following paragraphs, we will review some of the extant relevant studies addressing similar objectives to those formulated herein. For example, our findings differ from those of Koopmans et al who in a multicenter trial (HYPITAT) in Holland studied women with either gestational hypertension or mild preeclampsia who presented between 36 and <42 weeks' gestation. The subjects in this trial were prospectively randomized to either expectant management or induction of labor. They found that those allocated to induction of labor had better maternal outcomes compared with those allocated to expectant management (31 vs. 44%; *p* < 0.0001). 14 However, contrary to our study, Koopmans et al did not separate gestational hypertension from mild preeclampsia, making it unclear whether these two different conditions result in similar morbidities. In addition, while the Dutch study separated maternal morbidities by individual weeks of delivery, they combined neonatal morbidities into a single group with a median gestational age at induction of 38.7 weeks (range: 37.9–39.8), which may be why they did not detect a difference in neonatal outcomes. 14

A more recent multicenter, randomized trial by Magee et al randomized women with gestational and chronic hypertension during pregnancy to be delivered at 38 ^0/7–3/7^ weeks or receive “usual care at term.” They did not find any difference in maternal or neonatal morbidities. 15 However, this study included mothers with both chronic and gestational hypertension, most of whom were on antihypertensive medication during pregnancy (over 75% of mothers in both groups). In addition, there was minimal difference between the median gestational age at delivery between their groups (38.0 vs. 38.3) compared with our cohorts (37.0 vs. 39.0). Such differences likely account for our dissimilar results. Furthermore, this prospective study did not examine whether further delaying delivery to 39 or 40 weeks would have detected a beneficial effect that was otherwise missed by the study design or by restricting the delayed delivery planning to 38 weeks.

In contradistinction, our study findings concur with those of the large, multicenter study by Cruz et al who demonstrated that induction of labor between 38 and 39 weeks' balances the lowest maternal and neonatal morbidity and mortality. 16 To our knowledge, this is the first investigation that supports the results of Cruz et al questioning the justification for induction of labor at 37 weeks for gestational hypertension. However, given the limitations of our study, further research is still needed to address this important question.

In addition to being a retrospective, small study undertaken at a single institution, this study has other limitations. We could not ascertain the number of elevated BP measurements used to diagnose gestational hypertension or the gestational age at which the diagnosis was reached by the treating obstetrician. Multiple elevated BP readings might have a different prognosis than a limited number, and time of gestational presentation may also influence the risk of complications. Indeed, some of the mothers included in both cohorts were not diagnosed with gestational hypertension until they presented for delivery. The selection bias caused by including mothers diagnosed later in gestation may have influenced our results. In addition, due to the relatively small cohort sizes, we combined maternal morbidities in cohort 2 as 38 to 40 weeks, even though larger sample sizes might have discerned differences between 38 to 39, 39 to 40, and 40 to 41 weeks as previously shown in larger studies. 14 16 Furthermore, the unique health characteristics of our patient population may limit the applicability of our findings to other populations. The rate of gestational hypertension found in our study population was 11.4%, which is slightly higher than that currently reported in the literature. While this may partially be due to the increasing prevalence of gestational hypertension nationally, 4 the maternal health characteristics of our region are likely to also contribute to this discrepancy. West Virginia ranks second in maternal obesity, which has been shown to have an increased risk of hypertensive disorders associated with pregnancy. 17 Lastly, we only examined short-term morbidities in this study; there are reports assessing long-term neurodevelopmental delays in late preterm infants that could have further supported expectant management over delivery at 37 weeks. 18 19 20

Although our study had several limitations, one important strength was the systematic detection of gestational hypertension utilizing the electronic hospital database followed by extraction of all other clinical information for each of the cases from direct chart review of maternal and neonatal hospital records. This approach allowed for detailed monitoring of the progression of maternal disease to preeclampsia even beyond delivery in both cohorts and to confirm that no pregnancies resulted in stillbirths or maternal deaths.

While our study limitations render us unable to draw a conclusion regarding optimal timing of delivery for mothers with gestational hypertension, our findings do suggest that further investigation on this topic is warranted. We found that for women with gestational hypertension, delivery at 38 to 40 weeks was not associated with increased maternal morbidity but was associated with fewer neonatal complications and NICU admissions when compared with delivery at 37 weeks. However, it is unclear if the timing of gestational hypertension presentation influenced our results. As such, this study highlights the need for a prospective multicenter randomized control trial to evaluate if delivery induction beyond 37 weeks' gestation is a safe and beneficial option among women with gestational hypertension diagnosed prior to this gestational age to balance maternal and fetal risks.

## References

[1] Unger T Borghi C Charchar F 2020 International Society of Hypertension global hypertension practice guidelines J Hypertens 2020 38 06 982 1004 32371787 10.1097/HJH.0000000000002453

[2] Hutcheon J A Lisonkova S Joseph K S Epidemiology of pre-eclampsia and the other hypertensive disorders of pregnancy Best Pract Res Clin Obstet Gynaecol 2011 25 04 391 403 21333604 10.1016/j.bpobgyn.2011.01.006

[3] Abalos E Cuesta C Grosso A L Chou D Say L Global and regional estimates of preeclampsia and eclampsia: a systematic review Eur J Obstet Gynecol Reprod Biol 2013 170 01 1 7 23746796 10.1016/j.ejogrb.2013.05.005

[4] Ford N D Cox S Ko J Y Hypertensive disorders in pregnancy and mortality at delivery hospitalization - United States, 2017-2019 MMWR Morb Mortal Wkly Rep 2022 71 17 585 591 35482575 10.15585/mmwr.mm7117a1 PMC9098235

[5] Thornton J Duley L Gestational hypertension before term: observe or deliver? Lancet 2015 385 9986 2441 2443 25817376 10.1016/S0140-6736(14)62454-5

[6] Duley L The global impact of pre-eclampsia and eclampsia Semin Perinatol 2009 33 03 130 137 19464502 10.1053/j.semperi.2009.02.010

[7] Steegers E AP von Dadelszen P Duvekot J J Pijnenborg R Pre-eclampsia Lancet 2010 376 9741 631 644 20598363 10.1016/S0140-6736(10)60279-6

[8] Khan K S Wojdyla D Say L Gülmezoglu A M Van Look P F WHO analysis of causes of maternal death: a systematic review Lancet 2006 367 9516 1066 1074 16581405 10.1016/S0140-6736(06)68397-9

[9] Cunningham F G Leveno K J Bloom S L Pregnancy hypertension 23 ^rd^ ed. New York McGraw-Hill Professional 2009

[10] Report of the National High Blood Pressure Education Program Working Group on high blood pressure in pregnancy Am J Obstet Gynecol 2000 183 01 S1 S22 10920346

[11] Barton J R O'brien J M Bergauer N K Jacques D L Sibai B M Mild gestational hypertension remote from term: progression and outcome Am J Obstet Gynecol 2001 184 05 979 983 11303208 10.1067/mob.2001.112905

[12] Gestational hypertension and preeclampsia: ACOG Practice Bulletin, number 222 Obstet Gynecol 2020 135 06 e237 e260 32443079 10.1097/AOG.0000000000003891

[13] International Society for the Study of Hypertension in Pregnancy (ISSHP) Brown M A Magee L A Kenny L C The hypertensive disorders of pregnancy: ISSHP classification, diagnosis & management recommendations for international practice Pregnancy Hypertens 2018 13 291 310 29803330 10.1016/j.preghy.2018.05.004

[14] HYPITAT study group Koopmans C M Bijlenga D Groen H Induction of labour versus expectant monitoring for gestational hypertension or mild pre-eclampsia after 36 weeks' gestation (HYPITAT): a multicentre, open-label randomised controlled trial Lancet 2009 374 9694 979 988 19656558 10.1016/S0140-6736(09)60736-4

[15] WILL Trial Study Group Magee L A Kirkham K Tohill S Determining optimal timing of birth for women with chronic or gestational hypertension at term: the WILL (When to Induce Labour to Limit risk in pregnancy hypertension) randomised trial PLoS Med 2024 21 11 e1004481 39591427 10.1371/journal.pmed.1004481 PMC11593758

[16] Cruz M O Gao W Hibbard J U What is the optimal time for delivery in women with gestational hypertension? Am J Obstet Gynecol 2012 207 03 2140 2.14E8 10.1016/j.ajog.2012.06.009 22831812

[17] Addicott K Nudelman M Putty K Adverse perinatal outcomes associated with increasing maternal obesity Am J Perinatol 2024 41 09 1275 1281 37286181 10.1055/a-2107-1585 PMC11188758

[18] Adams-Chapman I Neurodevelopmental outcome of the late preterm infant Clin Perinatol 2006 33 04 947 964, abstract xi 17148014 10.1016/j.clp.2006.09.004

[19] Srinivas Jois R Neurodevelopmental outcome of late-preterm infants: a pragmatic review Aust J Gen Pract 2018 47 11 776 781 31207675

[20] Woythaler M Neurodevelopmental outcomes of the late preterm infant Semin Fetal Neonatal Med 2019 24 01 54 59 30322826 10.1016/j.siny.2018.10.002

